# Immediate chest tube removal at the completion of anterior vertebral tethering is well-tolerated without an increased risk of pulmonary complication

**DOI:** 10.1007/s43390-025-01132-w

**Published:** 2025-06-25

**Authors:** John T. Braun, Sofia C. Federico, Cornelia Griggs, David M. Lawlor, Daniel P. Croitoru, Brian E. Grottkau

**Affiliations:** 1 Massachusetts General Hospital, 55 Fruit St., Yawkey 3E, Boston, MA 02114 USA; 2 Massachusetts General Hospital, Boston, MA USA; 3 Massachusetts General Hospital, Harvard Medical School, Boston, MA USA; 4 Dartmouth Hitchcock Medical Center, Geisel School of Medicine at Dartmouth, Lebanon, NH USA

**Keywords:** Anterior vertebral tethering, Adolescent idiopathic scoliosis, Chest tube removal, Pulmonary complication

## Abstract

**Introduction:**

Though chest tube removal at the completion of an endoscopic thoracic procedure is well accepted in the pediatric and adult general surgery literature, this practice has never been studied in pediatric patients treated with anterior vertebral tethering (AVT) for AIS. This study retrospectively analyzed pulmonary complications in a large series of AIS patients consecutively treated with chest tube removal at the completion of AVT. The rate of pulmonary complication in this series was then compared with the published rate of pulmonary complication in patients managed with chest tube retention after AVT.

**Methods:**

A retrospective review of all AIS patients treated with AVT over a twelve year period yielded 257 consecutive patients (248 primary/9 revision) with 349 curves. Out of a total of 349 chest tubes placed intraoperatively, as a routine step of the procedure, 323 were removed at procedure completion while 26 were maintained for 2–5 days post-operatively as warranted. Patient charts, radiographs, and CT scans were reviewed to confirm any pulmonary complications.

**Results:**

In 257 AIS patients treated with AVT, 233 had chest tube removal at the completion of AVT with 4 (1.7%) peri-operative and 8 (3.4%) delayed pulmonary complications. Peri-operative complications included one symptomatic pneumothorax noted in the operating room that required chest tube reinsertion; one static pneumothorax that resolved without intervention; and two significant pleural effusions that resolved over time without intervention. Delayed complications included seven pleural effusions that occurred 2–6 weeks post-operatively and one chylothorax that occurred 1 week post-operatively. Several clinically significant pleural effusions (4/7) required thoracentesis or chest tube drainage but subsequently resolved. The chylothorax required chest tube drainage, dietary fat restriction, and treatment with octreotide. In 24 patients, 26 chest tubes were retained for 2–5 days post-op for a persistent air leak with presumed parenchymal injury (14), revision with significant adhesions (6), bleeding disorder (2), or diaphragmatic repair related to renal eventration (1) or congenital diaphragmic hernia (1).

**Conclusion:**

This study demonstrated the relative safety of immediate chest tube removal at the completion of AVT in AIS patients. The rate of pulmonary complication in 233 patients with chest tube removal at the completion of AVT was 5.1% which compared favorably with a published rate of 10–11% after chest tube retention. In 24 patients with an indication for chest tube retention at the completion of AVT, chest tube retention for 2–5 days resulted in no pulmonary complications.

## Introduction

Anterior vertebral tethering (AVT) is a minimally invasive alternative to fusion surgery for adolescent idiopathic scoliosis (AIS) that offers the potential for definitive scoliosis treatment. Benefits of AVT include the possible preservation of growth, motion, function, and overall health of the spine. As most AVT procedures involve access to the spine through the thorax, chest tube insertion is typically required intra-operatively to evacuate air, fluid, and blood from the pleural cavity prior to closure of the chest. However, despite the almost universal use of chest tubes at the completion of this procedure, there is significant variability in chest tube management among surgeons who perform AVT. While many surgeons prefer chest tube retention for several days post-operatively, with the goal of safe post-operative recovery of lung function, indwelling chest tubes may cause significant pain and decrease mobilization post-operatively [1].

Chest tube removal at the completion of AVT offers the possibility of decreased post-operative pain, improved patient mobilization, and even reduced hospital length of stay. These advantages must be balanced with the potential risk of pulmonary complication necessitating chest tube replacement post-operatively. Although significant support for chest tube removal at the completion of an endoscopic thoracic procedure exists in the pediatric and adult general surgery literature, this practice, to our knowledge, has never been studied in AIS patients treated with AVT [2–4]. The purpose of this study was to evaluate outcomes in a large consecutive series of AIS patients treated over 12 years with chest tube removal at the completion of AVT. Our hypothesis was that chest tube removal at the completion of AVT would be well-tolerated without an increased risk of pulmonary complication.

## Methods

**Fig. 1:**
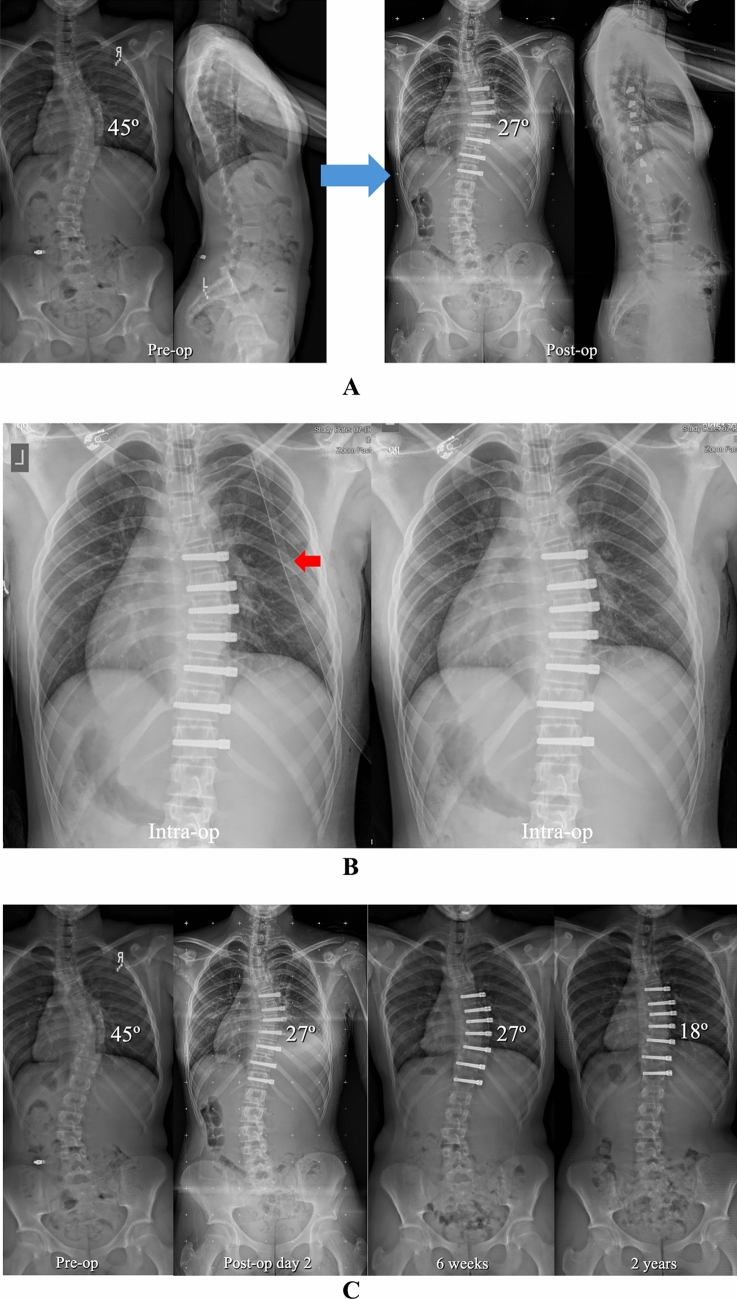
**A** Pre-operative and post-operative posteroanterior and lateral radiographs in a 13 + 5-year-old pre-menarchal female with AIS involving a progressive 45° curve right T6–T11 (Lenke 1B) at Risser 0 and Sanders 4. AVT was performed right T6–T12 via an endoscopic approach with lung isolation and uneventful chest tube removal at the completion of the procedure. Though a small right pleural effusion was noted on the post-operative radiograph, the patient was asymptomatic with no dyspnea, no oxygen requirement, and clear lung fields by auscultation. Therefore, no additional chest radiographs were performed. **B** Intra-operative chest radiographs before and after uneventful right chest tube (red arrow) removal with no evidence of pulmonary complication. **C** Pre-operative, post-operative, 6 weeks, and 2 year posteroanterior radiographs demonstrating resolution of the small right pleural effusion by 6 weeks and no pulmonary complication out to 2 years

**Fig. 2:**
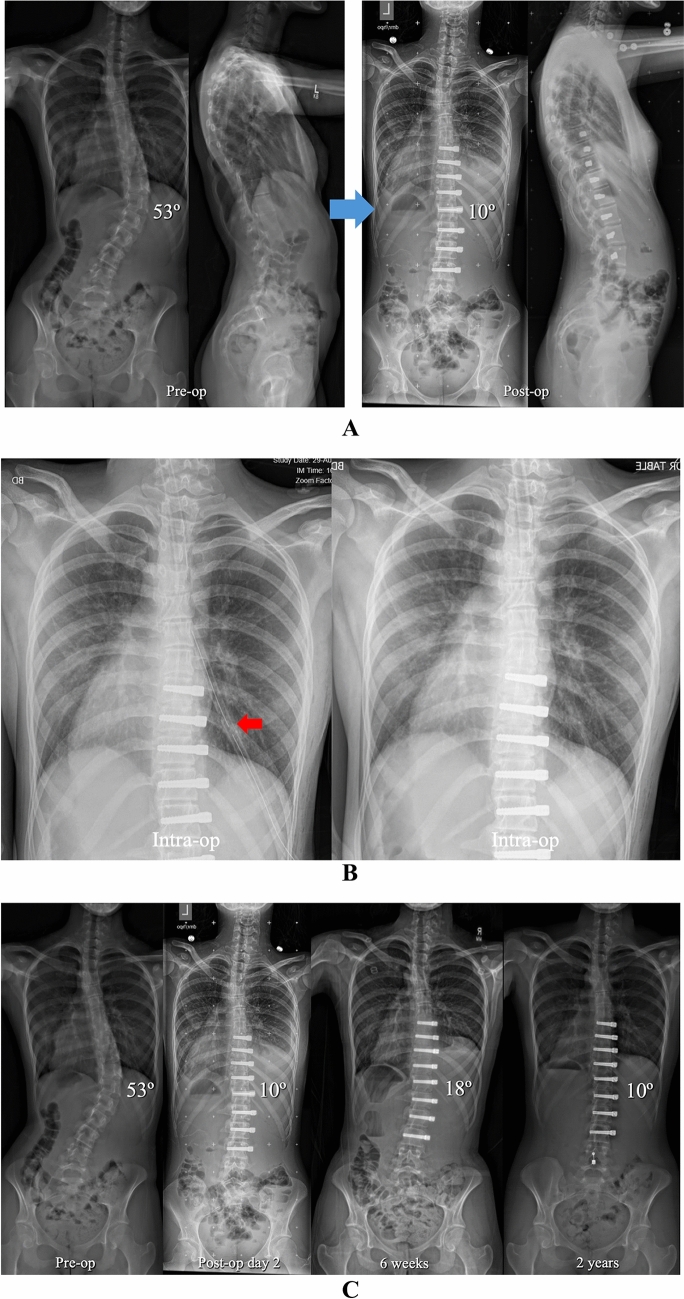
**A** Pre-operative and post-operative posteroanterior and lateral radiographs in a 14 + 5-year-old female with AIS involving a progressive 53° curve right T8–L2 (Lenke 5C) at Risser 0, Sanders 6, and 1.5 year post-menarchal. AVT was performed right T8–L3 via a mini open thoracoabdominal approach without lung isolation and with uneventful chest tube removal at the completion of the procedure. Though a small right pleural effusion was noted on the post-operative radiograph, the patient was asymptomatic with no dyspnea, no oxygen requirement, and clear lung fields by auscultation. Therefore, no additional chest radiographs were performed. **B** Intra-operative chest radiographs before and after uneventful right chest tube removal with no evidence of pulmonary complication. **C** Pre-operative, post-operative, 6 weeks, and 2 year posteroanterior radiographs demonstrating improvement of the small right pleural effusion by 6 weeks with full resolution over time

Under IRB approved protocols, a retrospective analysis was performed on all AIS patients consecutively treated with AVT from 2012 to 2024. The overall range of curve magnitude was 33–77° with skeletal maturity spanning Risser -1 to 5 (Risser -1 indicating Risser 0 with open triradiate cartilages) and Sanders 2–8. Both single and double curve patterns were treated in the thoracic and thoracolumbar/lumbar spine spanning Lenke types 1, 2, 3, 5, and 6. Charts and radiographs were reviewed to establish basic demographic data, confirm the details of chest tube management, and identify pulmonary complications in the first 90 days after surgery. Pulmonary complications were characterized as peri-operative if they occurred in the hospital and as delayed if they occurred after discharge. In addition to standard pre-operative and post-operative posteroanterior and lateral full length scoliosis radiographs, intraoperative chest radiographs were also performed before and after chest tube removal with additional post-operative chest radiographs as needed (Figs. 1, 2). CT scans were obtained in all patients with a significant pulmonary complication. The Cobb method was used to measure curve magnitude on pre-operative, post-operative, and final radiographs. Skeletal maturity was assessed using the Risser sign with an additional hand film to assess bone age as warranted.

The surgical technique for this procedure has been described previously by our group [5–7]. All patients were placed in a lateral decubitus position for surgery. A 3-portal endoscopic approach with single lung ventilation was used for all thoracic curves as well as for the rare thoracic dominant thoracolumbar curve spanning T7–T8 to L3–L4. A mini-open thoracoabdominal approach without lung isolation was used for the majority of thoracolumbar/lumbar curves spanning T9–T12 to L3–L4. Though the majority of the diaphragm was preserved in all cases, a small portion of the anterior and lateral diaphragm was usually released from the undersurface of the 11th rib to improve access to the lumbar spine. This portion of the diaphragm was usually repaired at the completion of the procedure. In addition, the crus of the diaphragm was minimally released medially and posteriorly over the T12 vertebra to allow tunneling of the cord or cords beneath the diaphragm from the lower thoracic to the upper lumbar levels. Segmental vessels were sacrificed prior to bicortical screw placement under fluoroscopic guidance. All levels of a given curve were instrumented with a polyethylene–terephthalate (PET) cord spanning Cobb end vertebra to end vertebra. The PET cord was then tensioned under fluoroscopic guidance to achieve the desired correction of disc angulation at each level with the typical goal of achieving a neutral disc. Tensioning proceeded from proximal to distal with maximal tension applied to the apical segments, with less tension at the ends. A temporary 24 French chest tube was used to assist with reinflation of the lung and evacuation of blood and fluid from the chest but was then removed, under appropriate circumstances, at the end of the procedure. These appropriate circumstances included no evidence of pneumothorax, hemothorax, or fluid collection on an intraoperative chest X-ray; no Pleur-evac air leak to suggest a parenchymal lung injury; no significant risk of post-operative bleed due to a bleeding disorder; and no significant risk of post-operative effusion from a complex revision approach requiring take-down of multiple adhesions. When any of these circumstances were present, the chest tube was retained with a plan to remove it on post-operative days 2–5. Spinal cord monitoring was used in all cases. Patients with double curve patterns had both curves treated under one anesthetic with the thoracic curve treated first. An access surgeon was utilized for all procedures.

Hydroxyapatite coated titanium screws and a polyethylene–terephthalate (PET) cord from the Dynesys Dynamic Stabilization System (Zimmer Biomet Spine/ZimVie, Broomfield, CO) were used in all cases without the polycarbonate–urethane spacer during the first 8 years of the study. As this device system was approved by the FDA in 2003 for adult lumbar spine stabilization as an adjunct to fusion, its use in the treatment of scoliosis in children during this portion of the study time period was considered an off-label indication. During the last 4 years of the study, hydroxyapatite coated titanium screws and a polyethylene–terephthalate (PET) cord from The Tether System (ZimVie/Highridge, Westminster, CO) were used. This device system achieved FDA approval in 2019.

Statistical analysis was performed using paired and one-sided *t* tests, where appropriate, to determine the significance of pre-op versus post-op or final radiographic measurements. Chi squared analysis was used to determine the significance of variables associated with revision surgery. All statistical analyses were conducted in Microsoft Excel for Mac (version 16.52; Microsoft) with an alpha set at 0.05.

## Results

**Fig. 3:**
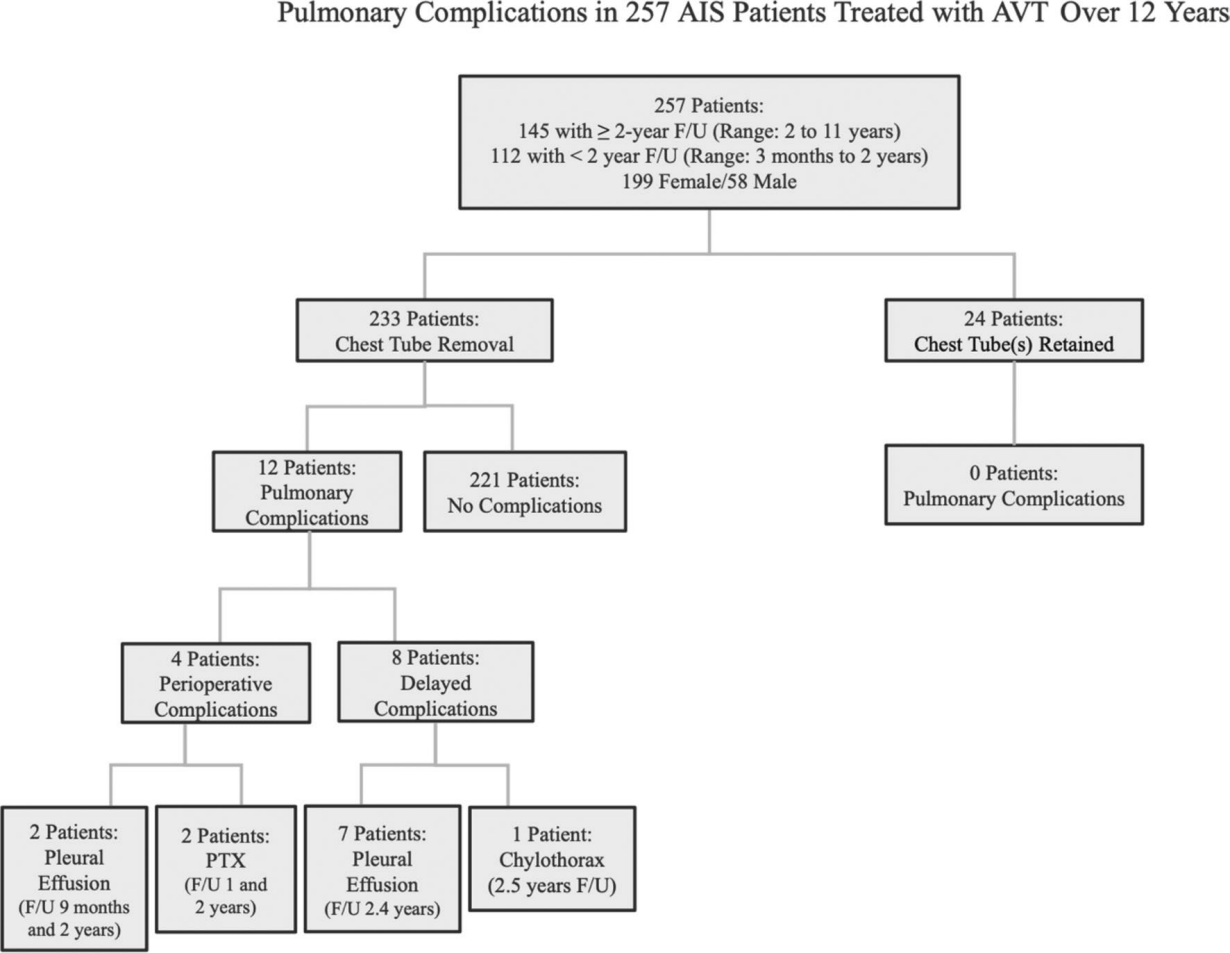
Pulmonary complications in 257 patients treated with AVT over 12 years

Two hundred fifty-seven consecutive AIS patients were treated with AVT for both single and double curve patterns with an average pre-operative curve magnitude of 50.2° at age 14.8 years and Risser 2.6/Sanders 5.2. All pulmonary complications occurred within the first 90 post-operative days with no pulmonary complications encountered after 90 days. This included the 112 patients with less than 2 year follow-up as well as the 145 patients with 2–11 year follow-up (Fig. 3).

**Fig. 4:**
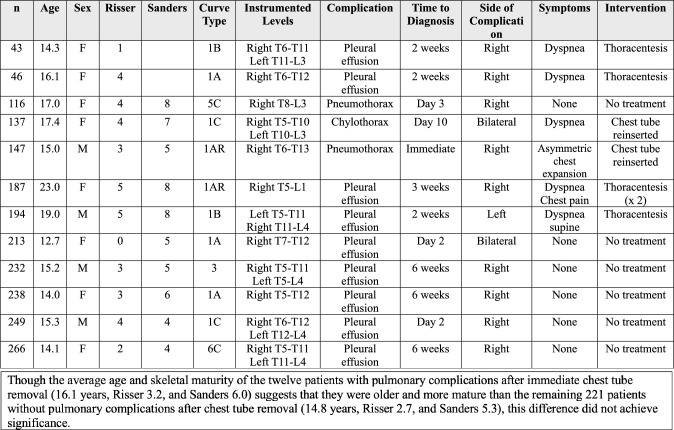
Presentation and management of pulmonary complications in AIS patients after AVT

**Fig. 5:**
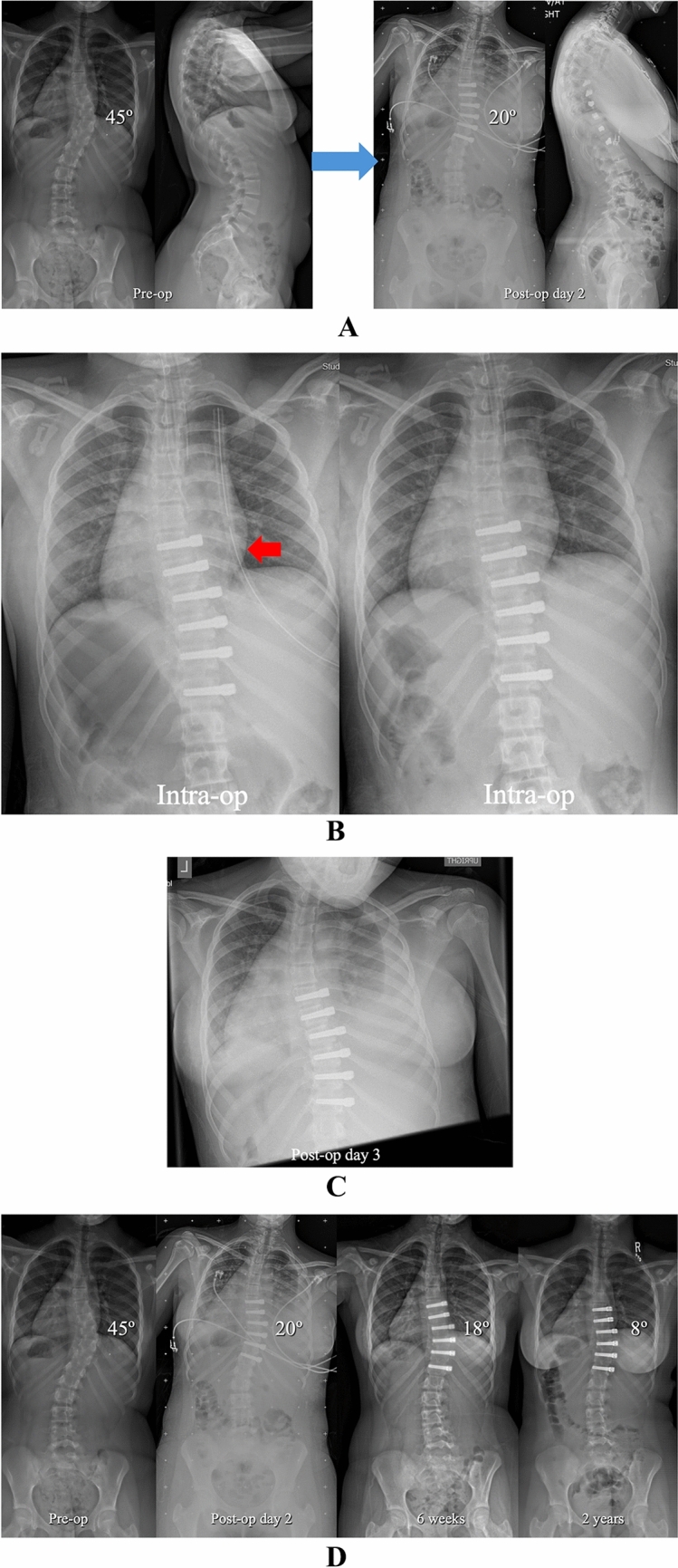
**A** Pre-operative and post-operative posteroanterior and lateral radiographs in a 12 + 8-year-old female with AIS involving a progressive 45° curve right T7–T12 (Lenke 1A) at Risser 0, Sanders 5, and 1 month post-menarchal. AVT was performed right T7–T12 via an endoscopic approach with lung isolation and with uneventful chest tube removal at the completion of the procedure. Though a moderate right pleural effusion was noted on the post-operative radiograph, the patient’s mild dyspnea with ambulation and oxygen requirement of 0.5–1.5L by nasal cannula resolved by post-operative day 3. **B** Intra-operative anteroposterior chest radiographs demonstrating uneventful chest tube removal with no evidence of pulmonary complication. **C** Portable anteroposterior chest radiograph on post-operative day 3 demonstrating a slight increase in the moderate pulmonary effusion and bibasilar opacities consistent with atelectasis. The patient, however, was asymptomatic at this time with no dyspnea, no oxygen requirement, and only slightly diminished breath sounds by auscultation. **D** Pre-operative, post-operative, 6 weeks, and 2 year posteroanterior radiographs demonstrating a moderate pleural effusion and bibasilar opacities consistent with atelectasis that resolved by 6 weeks with no recurrence of any pulmonary issue out to the 2 year post-operative timepoint

**Fig. 6:**
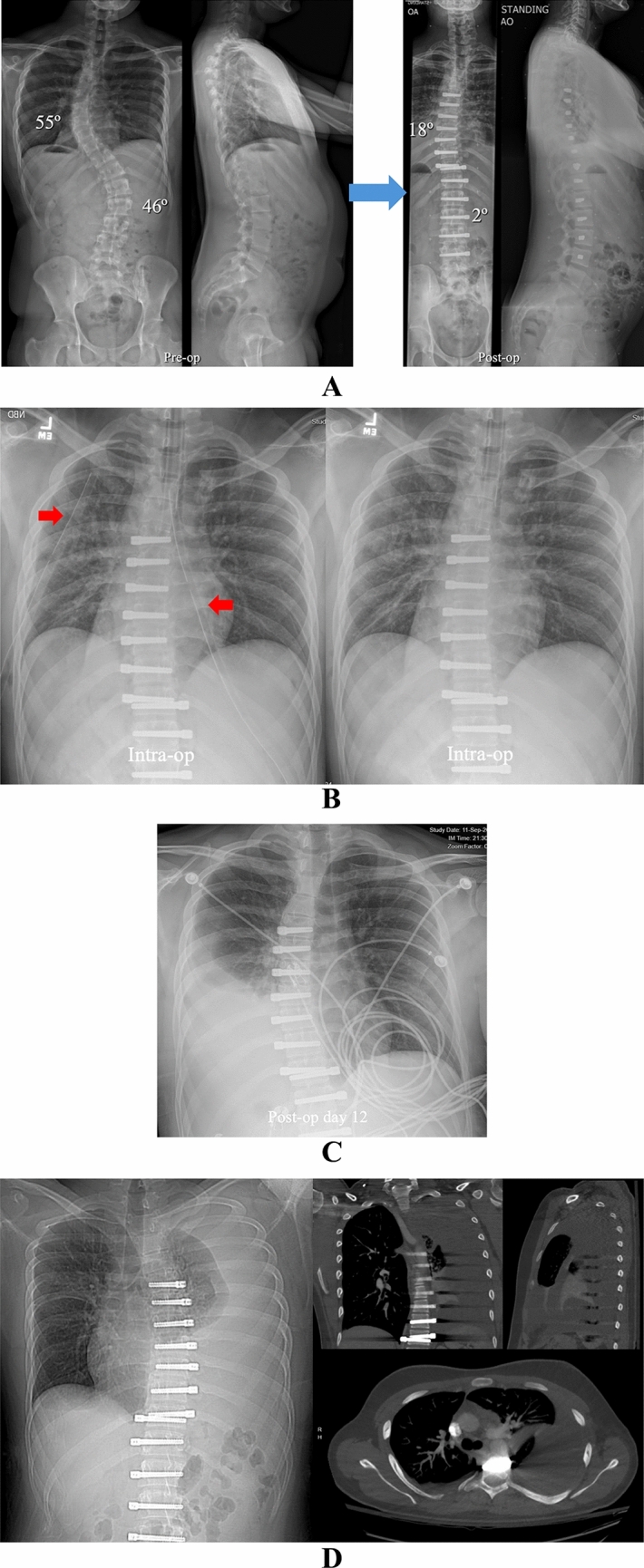

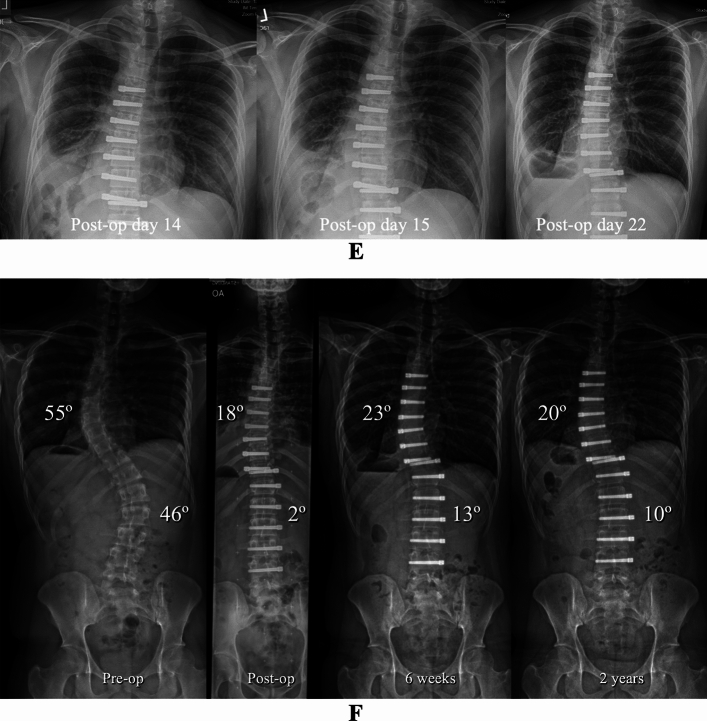
**A** Pre-operative and post-operative posteroanterior and lateral radiographs in a 19-year-old male with AIS involving 55° and 46° curves left T5–T11 and right T11–L4 (Lenke 1C), respectively, at Risser 5 and Sanders 8. AVT was performed right T5-T11 via an endoscopic approach with lung isolation and left T11-L4 via a mini open thoracoabdominal approach without lung isolation and with uneventful bilateral chest tube removal at the completion of both procedures. Though the post-operative posteroanterior radiograph is suboptimal, the post-operative lateral radiograph demonstrated a mild pleural effusion in this asymptomatic patient. **B** Intra-operative anteroposterior chest radiographs demonstrating uneventful bilateral chest tube removal with no evidence of pulmonary complication. **C** Portable chest radiograph demonstrated a moderate pleural effusion on post-operative day 12 after the patient presented to the emergency department with complaints of fatigue, lightheadedness, and diarrhea. **D** CT scan on post-operative day 13 demonstrated an increase in the large pleural effusion. Subsequent thoracentesis allowed evacuation of 2085 cc of serosanguinous fluid. **E** Portable chest radiographs on post-operative days 14, 15, and 22 demonstrating gradual resolution of the pleural effusion over time after thoracentesis. **F** Pre-operative, post-operative, 6 weeks, and 2 year posteroanterior radiographs demonstrating progressive resolution of the left pleural effusion over time

Of the 233 patients that were managed with immediate chest tube removal at the completion of AVT, 1.7% (4/233) had peri-operative pulmonary complications and 3.4% (8/233) had delayed pulmonary complications (Fig. 4). The peri-operative complications included one symptomatic pneumothorax, noted in the operating room, which required chest tube reinsertion without sequelae. This chest tube was removed uneventfully on post-operative day 2. Three additional patients with asymptomatic pulmonary issues, one static pneumothorax and two significant plural effusions, were noted post-operatively, while these patients were still in the hospital but resolved with appropriate monitoring without the need for intervention (Fig. 5). The delayed complications included seven patients who developed a significant pleural effusion 2–6 weeks post-operatively and one patient who developed a chylothorax 1 week post-operatively. The majority of the patients with a significant delayed pleural effusion (4/7) were symptomatic and required thoracentesis or chest tube drainage (Fig. 6) with subsequent resolution, while the remainder (3/7) were asymptomatic and resolved with appropriate monitoring. The single patient with a chylothorax demonstrated progressive dyspnea and required 5 days of chest tube drainage as well as dietary fat restriction and octreotide.

In the 24 remaining patients with chest tube retention, 26 of 34 chest tubes were retained for 2–5 days post-operatively. Chest tubes were intentionally maintained for clinical indications including evidence of a persistent air leak on the Pleur-evac, presumed to represent an inadvertent parenchymal lung injury (14); a complex revision approach requiring take-down of significant adhesions with concern for possible parenchymal lung injury (6); a bleeding disorder (2); and diaphragmatic repair in one patient related to renal eventration and in another patient with a congenital diaphragmic hernia. None of the patients with chest tube retention demonstrated peri-operative or delayed pulmonary complications.

All pulmonary complications in this study occurred in patients with both single and double curve patterns (Lenke 1A, 1B, 1C, 3, 6) in which a thoracic or thoracolumbar curve was treated endoscopically and required intra-operative lung isolation. No pulmonary complications occurred in patients with a single thoracolumbar curve pattern (Lenke 5) treated via a mini-open procedure without lung isolation. All pleural effusions and pneumothoraces occurred in the pleural cavity treated with a thoracoscopic approach to the thoracic or thoracolumbar curve. The single chylothorax was the only complication that did not occur ipsilateral to thoracoscopic access. Instead, it developed in the contralateral chest after treatment of a second curve via a mini open thoracoabdominal approach without lung isolation. Pleural effusion was the most common complication (9/12) and was most often delayed (7/9), occurring 2–6 weeks post-operatively. Patients with a delayed pleural effusion were more likely to require thoracentesis or chest tube drainage (4/7) than those with a peri-operative pleural effusion (0/2). All symptomatic pleural effusions required treatment.

## Discussion

In this study, the relative safety of immediate chest tube removal at the completion of AVT was demonstrated in a large consecutive series of AIS patients treated over a 12 year period. The rate of pulmonary complication in 233 patients with chest tube removal at the completion of AVT was 5.1% (12/223) which compared favorably with a reported pulmonary complication rate of 10–11% in AVT patients with chest tube retention [10, 11]. Similar to these published reports, the majority of the pulmonary complications in this study were delayed (8/12), involved pleural effusion (7/8), and were successfully treated with thoracentesis or chest tube drainage. The additional complication of chylothorax was rare in this study as well as in other published reports but required chest tube drainage and additional treatment measures. The peri-operative pulmonary complications of pneumothorax, hemothorax, and significant pleural effusion were uncommon in all studies, with no occurrence of hemothorax in this study.

Chest tube retention at the completion of AVT was indicated in 24 patients for findings of an air leak on evaluation of the Pleur-evac at procedure completion, presumed to be related to an inadvertent parenchymal lung injury (14/24). Chest tubes were also retained in patients thought to be at an increased risk for post-operative pleural effusion or hemothorax. Circumstances thought to be associated with an increased risk of pulmonary or pleural complication included patients requiring a complex revision approach with takedown of significant pulmonary adhesions (6/24) or a history of a bleeding disorder (2/24).

Two unique cases required chest tube retention after diaphragmatic repair. In the first case, a region of diaphragmatic eventration from a displaced left kidney required relocation of the kidney into the abdomen, excision of a patulous portion of the diaphragm, and then imbrication of the diaphragm to repair the defect. In the second case, a congenital diaphragmatic hernia required standard repair. In all patients with chest tube retention, chest tube removal after 2–5 days was uneventful with no subsequent pulmonary complications.

Though immediate chest tube removal at the completion of AVT has not, to our knowledge, been previously studied, this method of chest tube management has gained increasing acceptance in both pediatric and adult thoracic and general surgery (1–4). New protocols focusing on enhanced recovery after surgery (ERAS) have been applied to thoracic surgery following success with similar protocols in colorectal surgery [8]. The pediatric and adult thoracic surgery literature has even begun to explore the avoidance of post-operative chest tubes for more invasive thoracic procedures, such as partial lung resections and lobectomies [8, 9]. The rationale for the avoidance of prolonged use of chest tubes is primarily to improve post-operative pain and thereby facilitate safe and effective mobilization after surgery. While patients benefit directly from the reduction in post-operative pain and the increased efficiency of mobilization without a chest tube in place after a major surgical intervention, there may be additional advantages to immediate chest tube removal via a reduction in the complexity of post-operative care and in the overall length of the hospital stay. For these reasons, it would be beneficial to focus future studies on the specific evaluation of these patient centered and system-related outcomes.

The major limitations of this study are the retrospective nature of the analysis and the absence of a control group for comparison. For example, while there were no pulmonary complications after chest tube retention in this study, these patients represented only a small fraction of the patients treated and, therefore, were not ideal as a comparison group. For this reason, no statistical significance could be demonstrated in this study between the two groups treated with chest tube removal versus retention. In the future, a prospective, randomized study of AVT patients managed with chest tube removal versus retention would likely provide robust data to assess the safety and efficacy of immediate versus delayed chest tube removal after AVT. A prospective study would not only allow analysis of peri-operative and delayed pulmonary complications after AVT in patients with chest tube retention versus removal but would also allow an assessment of patient centered outcomes to drive interventions for optimal patient recovery.
